# Preventing relapse in non-infectious uveitis affecting the posterior segment of the eye – evaluating the 0.2 μg/day fluocinolone acetonide intravitreal implant (ILUVIEN^®^)

**DOI:** 10.1186/s12348-020-00225-z

**Published:** 2020-11-30

**Authors:** Bahram Bodaghi, Quan Dong Nguyen, Glenn Jaffe, Ramin Khoramnia, Carlos Pavesio

**Affiliations:** 1 Department of Ophthalmology, IHU FOReSIGHT, Sorbonne-APHP, Paris, France; 2 Byers Eye Institute, Spencer Center for Vision Research, Stanford University of Medicine, Palo Alto, California USA; 3 Department of Ophthalmology, Duke University, Durham, North Carolina USA; 4 International Vision Correction Research Centre (IVCRC), The David J. Apple International Laboratory for Ocular Pathology, Department of Ophthalmology, Ruprecht-Karls-University of Heidelberg, Heidelberg, Germany; 5 Moorfields Eye Hospital, London, UK

**Keywords:** Non-infectious uveitis, Posterior segment of eye, Recurrence, Intravitreal, Corticosteroid, Fluocinolone acetonide, Implant, Microdosing, Macular oedema, Chronic

## Abstract

**Background:**

The current article is a short review of an Alimera Sciences-sponsored symposium held during The 15th International Ocular Inflammation Society Congress in Taiwan on the 14th November 2019 entitled, ‘*Preventing relapse of non-infectious uveitis effecting the posterior segment of the eye – evaluating the 0.2 μg/day fluocinolone acetonide intravitreal implant*.’

**Main text:**

The fluocinolone acetonide intravitreal implant was approved in Europe for the prevention of relapse in recurrent non-infectious uveitis affecting the posterior segment of the eye and offers a systemic therapy-sparing treatment option by providing low daily dose of corticosteroid into the vitreous for up to 3 years. In the symposium, the presenters reported clinical outcomes from patients with non-infectious uveitis effecting the posterior segment of the eye to support the effectiveness and safety of the implant for up to 3 years in both randomised controlled trials and real-world practices.

**Conclusions:**

Data showed that over a 36 month period, treatment with the fluocinolone acetonide intravitreal implant was associated with significantly fewer episodes of uveitic recurrence, a significantly longer time to uveitic recurrence, greater improvement in visual acuity, a lower need for adjunctive therapy, and an acceptable safety profile.

## Background

This article provides a short review of an Alimera Sciences-sponsored symposium held during The 15th International Ocular Inflammation Society Congress in Taiwan on the 14th November 2019 and entitled, ‘*Preventing relapse of non-infectious uveitis effecting the posterior segment of the eye – evaluating the 0.2 μg/day fluocinolone acetonide intravitreal implant*.’ Professor Bahram Bodaghi provided an introduction to the symposium and this was followed by four talks. Professor Quan Dong Nguyen discussed the lessons learned in managing patients with uveitis and avoidance of cumulative damage; Professor Glenn Jaffe talked about the long term follow-up of an individual investigator-sponsored trial of fluocinolone implant to treat non-infectious intermediate, posterior and panuveitis; and, then Professor Ramin Khoramnia and Mr. Carlos Pavesio delivered presentations that focused on patient case studies. These talks are summarised in this article.

## Main text

### Introduction by Professor Bahram Bodaghi

Repeated inflammatory episodes in recurrent non-infectious uveitis can negatively impact vision and so adequate preventive treatment is essential to optimise visual outcomes. Although several treatment options are available, including corticosteroids and immunomodulatory therapy, until recently there have been no intravitreal treatments available in Europe offering sustained efficacy for more than 6 months. However, in 2019, a long-acting intravitreal implant designed to sustain efficacy for up to 3 years was approved in many European countries. This fluocinolone acetonide (FAc) implant (ILUVIEN^®^, Alimera Sciences, Hampshire, UK) achieves long-lasting effects through continuous microdose release of the corticosteroid. The implant is indicated for the prevention of relapse in recurrent non-infectious uveitis affecting the posterior segment of the eye (NIU-PS) [1]. Designed to release fluocinolone acetonide at a rate of 0.2 μg/day for up to 3 years, the implant facilitates continuous long-term protection against inflammation and so avoids the cycle of treat, recur and treat. By helping to prevent recurrences of NIU-PS, the implant ultimately helps protect and maintain vision for longer — with the added advantage of also requiring fewer injections and fewer clinic visits for treatment than shorter-acting implants.

The following presentations further discuss the use of the FAc implant in a variety of patient types.

### Avoiding cumulative damage in uveitis by Professor Quan dong Nguyen

The total insult to the uveitic eye from repeated inflammatory attacks may be greater than the sum of individual insults and the consequences can be irreversible. Thus, avoiding cumulative damage in uveitis by preventing recurrent episodes of inflammation is critical for minimising ocular damage. Other significant risks affecting visual outcomes in uveitis include a greater than 12-month delay between onset of symptoms and presentation to a uveitis subspecialist, and the development of glaucoma [2].

**Fig. 1:**
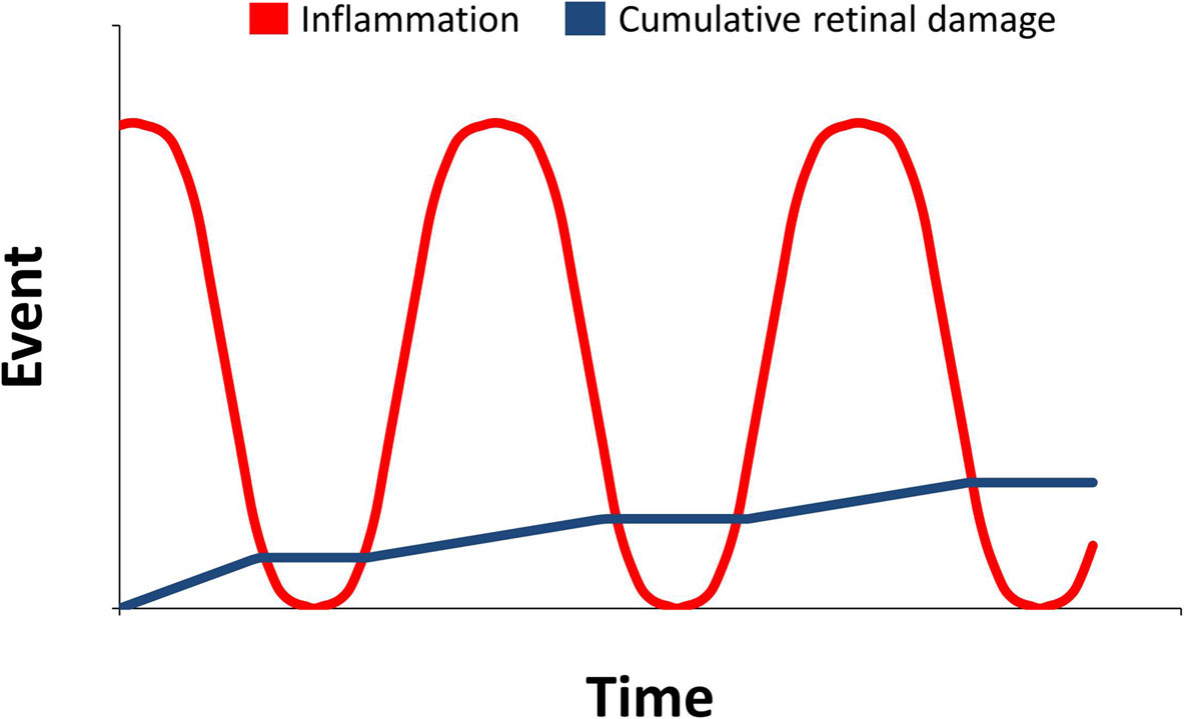
Inflammation that relapses and is then retreated leads to accumulative retinal damage and poorer visual outcomes [3]

Corticosteroids are the mainstay of treatment for NIU-PS and may be delivered orally, intravitreally or periocularly. However, many of these treatments are relatively short acting and small flares of uveitis are typically allowed to occur between corticosteroid injections. Such practice means that patients with recurrent uveitis who are treated in this manner typically suffer incremental declines in vision over a period of years that, ultimately, result in loss of vision (Fig. 1). Furthermore, it has been hypothesised that sustained control of inflammation results in better long-term visual outcomes than is achieved in eyes where inflammation is relapsing and being retreated, and that this may be due to fact that the total insult to the uveitic eye from repeated inflammatory attacks may be greater than the sum of individual insults [3].

**Fig. 2:**
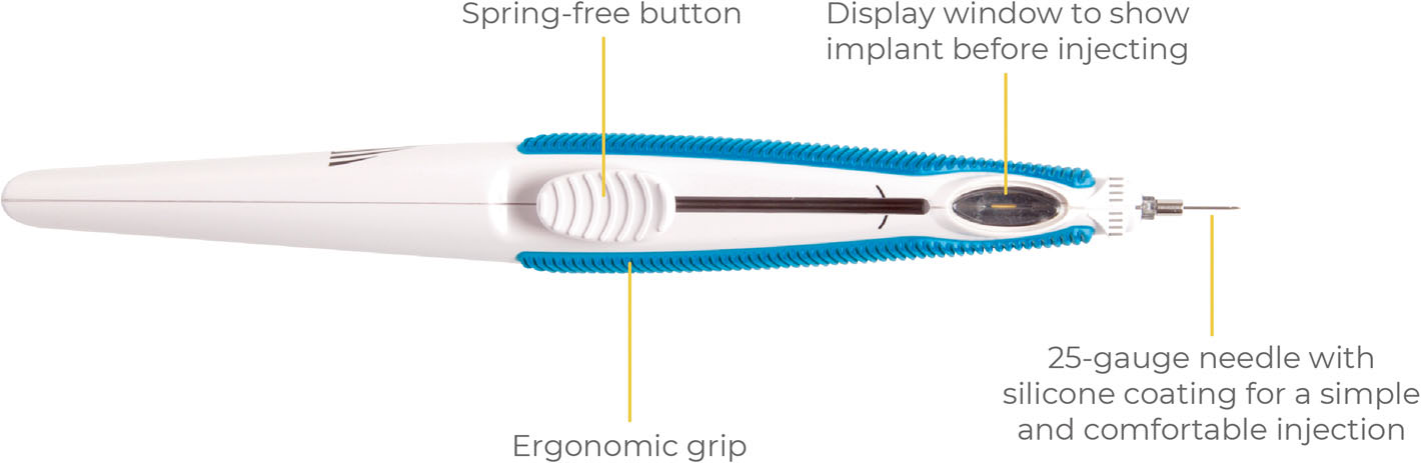
The 0.2 μg/day FAc intravitreal implant is a polyimide polymer tube that is injected intravitreally through a modified 25-gauge needle injector. The tube contains a 3 mm core of fluocinolone acetonide that is designed to release the corticosteroid within the vitreous for up to 36 months

As non-infectious uveitis is usually a chronic disease, therapeutic options often need to be as long lasting as possible in order to eliminate or at least minimise the number of flares occurring between treatments. The 0.2 μg/day FAc intravitreal implant is designed to offer efficacy in preventing intraocular inflammation for up to 3 years and is indicated for the prevention of relapse in recurrent NIU-PS (Fig. 2). Intravitreal delivery of corticosteroids offers a potentially improved safety profile relative to oral delivery (by minimising the potential for systemic adverse events), although showing this statistically [3] requires further study in a larger number of patients. Furthermore, long-lasting therapy additionally helps to minimise the number of flares.

A single intravitreal injection of the 0.2 μg/day FAc implant was evaluated over 36 months in a phase 3 study of patients with NIU-PS (PSV-FAI-001 [4]) where it was found to be associated with significant clinical benefits compared with a sham injection (Table 1) [5]. Specifically, the FAc implant group had significantly fewer episodes of uveitic recurrence in the 36 months post-implantation (mean of 1.7 versus 5.3, *p* < 0.001) and a significantly longer time to uveitis recurrence (median of 657.0 versus 70.5 days, p < 0.001) than the sham-treated group [5]. The FAc implant group also achieved a relatively greater mean (± standard deviation [SD]) improvement in best corrected visual acuity over 36 months — + 9.1 (± 13.0) Early Treatment Diabetic Retinopathy Study letters versus + 2.5 (± 14.2) in the sham group (*p* = 0.020) — and was associated with a lower likelihood of adjunctive topical or systemic treatments being used than in the sham group (57.5% versus 97.6% of patients).

**Table 1:** Key benefits and safety monitoring associated with the 0.2 μg/day FAc implant

Key Benefits	Safety Monitoring
• Sustained control of ocular inflammation for up to 36 months which, compared with a sham injection, offers :[4] • Fewer uveitis recurrences • Longer periods before recurrence • Less potential for cumulative damage from repeated cycles of inflammation • Greater improvement in visual acuity • Reduced need for adjunctive treatments • The potential to minimise systemic adverse events* [3] (plasma levels of fluocinolone acetonide are below the lower limit of quantitation [1])	• Raised intraocular pressure • Cataract development

**Notes:** *Intravitreal delivery of corticosteroids offers a potentially improved safety profile relative to oral delivery (by minimising the potential for systemic adverse events), although showing this statistically [3] requires further study in a larger number of patients. Long-lasting therapy additionally helps to minimise the number of flares

Intravitreal corticosteroid treatments are well known to increase the risk of raised intraocular pressure and to lead to cataract development (Table 1). The proportion of eyes requiring intra-ocular pressure (IOP)-lowering medication was 42.5% in the FAc implant group versus 33.3% in the sham group, whereas the proportion that underwent IOP-lowering surgery was 5.7% versus 11.9%, respectively. The FAc implant increased the need for cataract surgery to 73.8% of eyes in the implant group versus 23.8% in the sham group.

It is essential to realise the importance of appropriate patient selection when considering FAc implantation because, as shown in the guidance in Table 2, clinicians need to consider individual patient characteristics together with potential risks and benefits. The FAc implant is suitable for prevention of relapse in recurrent NIU-PS because, unlike shorter-acting treatments such as intravitreal triamcinolone or dexamethasone, the long-acting FAc implant can provide a continuous daily dose of corticosteroid for up to 3 years of treatment — and, importantly, such continuous daily dosing provides better protection against inflammatory episodes than repeated injections with a shorter-acting treatment. Thus, chronic uveitis is better managed with a long-acting treatment and acute uveitis is better managed with a shorter-acting treatment. Intravitreal implants (such as the FAc and dexamethasone implants) are also better suited than systemic treatment for treating patients with unilateral or asymmetric ocular disease as, being local treatments, they minimise the potential for risks associated with systemic treatment.

**Table 2:** Appropriate patient selection for the 0.2 μg/day FAc intravitreal implant

Appropriate Patients	Inappropriate Patients
• Uveitis expected to recur or persist for more than 2 years • Treatment decisions are driven by ocular disease • Non-responsive to, or intolerant of, standard therapy including systemic corticosteroids and various immunomodulatory therapeutic agents • Sight-threatening disease • Patients wishing to minimise the number of injections they receive (especially needle-phobic patients) • EITHER no previous elevation of intraocular pressure in response to steroids OR willing to have medication or surgery if needed to reduce intraocular pressure • Likely to comply with follow-up visits even when uveitis is quiet • Patients with unilateral or bilateral disease	• Acute disease (as long-lasting treatment not required) • Treatment decisions are NOT driven by ocular disease (e.g. if systemic treatment is still needed) • Glaucoma • Infectious uveitis • Any other condition masquerading as non-infectious uveitis • Not likely to comply with follow-up visits

#### Key point from presentation

Chronic or recurrent uveitis needs a long-acting treatment (rather than repeated short-acting treatments) acute uveitis may need to be controlled with a short-acting treatment initially.

### Evaluating the FAc implant in the treatment of uveitis by Professor Glenn Jaffe

Before the phase 3 study discussed above was performed, Professor Jaffe and colleagues conducted a prospective US Food and Drug Administration (FDA)-approved individual investigator-sponsored investigational new drug study and a long-term retrospective follow-up study of the 0.2 μg/day FAc intravitreal implant. Results from these studies complement the previously described phase 3 data and provide long-term follow-up data for up to 80 months post-implantation (more than 6 years).

Patients were eligible to enrol if they had had recurrent non-infectious uveitis (intermediate, posterior or panuveitis) for at least 1 year, 2 uveitis recurrences in the preceding 6 months and systemic corticosteroid or immunosuppressive therapy for at least 3 months. Exclusion criteria included systemic immunosuppressive therapy for non-ocular disease and glaucoma or steroid-responsive intraocular pressure in the absence of prior filtration surgery. Patients were initially randomised to receive one of two implant doses and were followed for at least 3 years. The lower dose FAc implant was equivalent to that later used in a phase 3 study (PSV-FAI-001 [4]) and subsequently marketed. The evaluation involved two individual investigator studies running sequentially — a 2-year dose-randomised, dose-masked, prospective study [6] followed by a retrospective, longitudinal follow-up study [7] of patients who were followed for at least 1 additional year after the end of the first study.

**Fig. 3:**
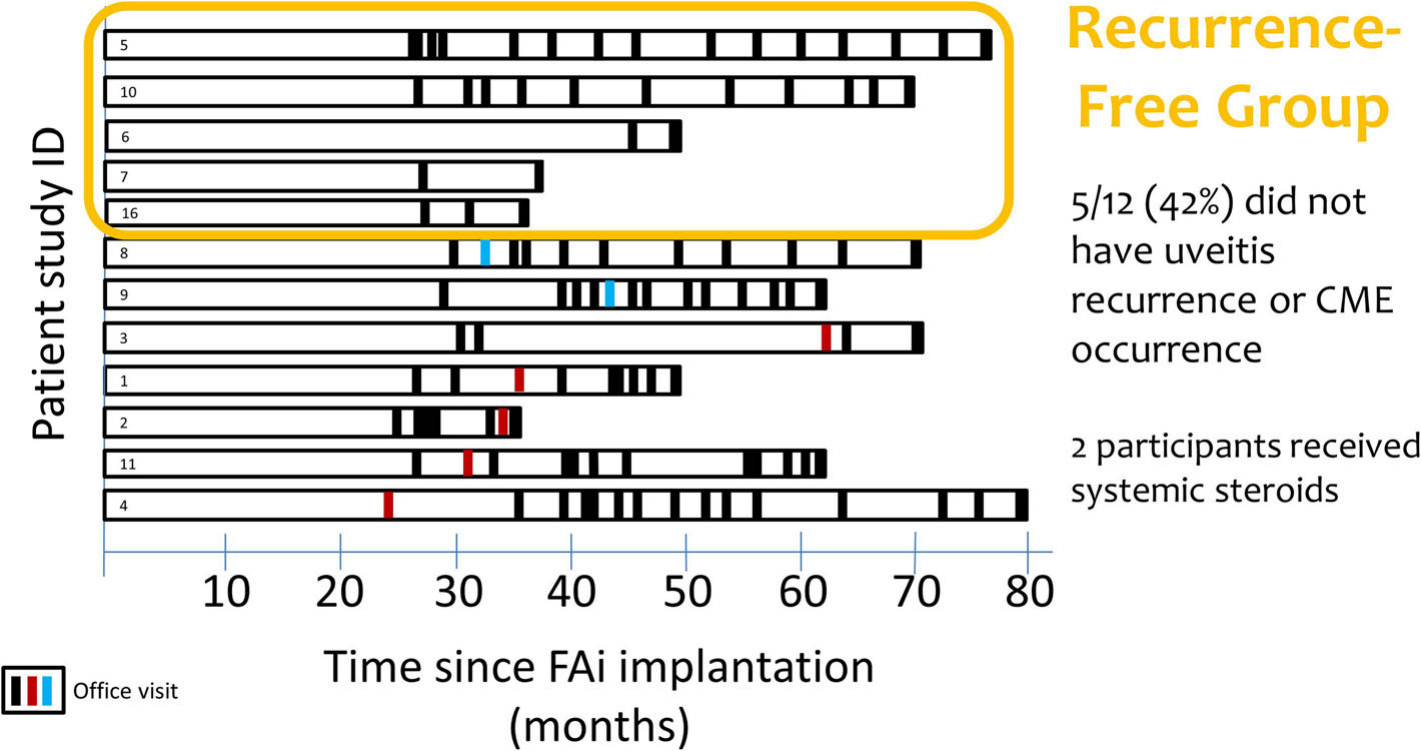
Among 12 patients who received a single 0.2 μg/day FAc  intravitreal implant, 5 (42%) did not experience a recurrence of either uveitis or cystic macular oedema during the follow-up period of between 36 and 80 months. Black lines indicate clinic visits, blue lines indicate recurrence of cystoid macular oedema, red lines indicate recurrence of uveitis

Overall, 12 patients were followed for at least 3 years and the maximum follow-up was 6 years and 8 months. The majority (83%) were female and all were either African American (50%) or white (50%). Most patients had intermediate uveitis together with anterior uveitis, or panuveitis. Their mean age was 43 years (range 25–64 years) – thus, many patients were in the prime years of their working lives. Among the 12 patients, 5 (42%) had no recurrence of uveitis or cystoid macular oedema during the follow-up period of between 36 and 80 months. The remaining 7 patients had a recurrence – 2 (17%) had a recurrence of cystoid macular oedema (at a mean of 36.9 months, range 36.1–42.1 months) and 5 (42%) had a recurrence of uveitis (at a mean of 36.1 months, range 22.8–61.1 months; or a mean of 29.8 months if a patient who had no clinic visits for 2 years before the reported recurrence is excluded) (Fig. 3). Therefore, although the FAc implant does not appear to be a curative treatment (though possibly the disease burns itself out in some patients), it offers excellent disease control and can prevent recurrences of inflammation over considerable periods of time. Indeed, the authors of a case report describing a patient with panuveitis who demonstrated sustained control of inflammation and macular oedema for at least 3 years after treatment with the FAc implant concluded that the “benefits may persist even after cessation of the direct anti-inflammatory effect of the implant” [8].

The mean visual acuity at the beginning and end of the follow-up study was 20/31.4 (presented as a Snellen fraction; range 20/20 to 20/640) and 20/37.7 (range 20/20 to 20/160), respectively. In terms of safety, 2 patients had an Ahmed glaucoma valve implant during the first study and 3 had a rise in intraocular pressure > 21 mmHg in the follow-up study that was controlled with drops (though 2/3 patients opted for incisional surgery in preference to having frequent drops). As would be anticipated with any intravitreal corticosteroid, both patients whose eyes were phakic during the first study formed cataracts within 2 years of implantation.

Overall, the results from these studies demonstrated that the FAc implant provided prolonged efficacy against recurrent non-infectious uveitis with an acceptable safety profile – and helped justify the rationale for progressing to a phase 3 trial.

#### Key point from presentation

42% of patients had no recurrence of uveitis or cystoid macular oedema during the follow-up period of between 36 and 80 months.

### Real-world data – case series in non-infectious uveitic macular oedema by Professor Ramin Khoramnia

**Fig. 4:**
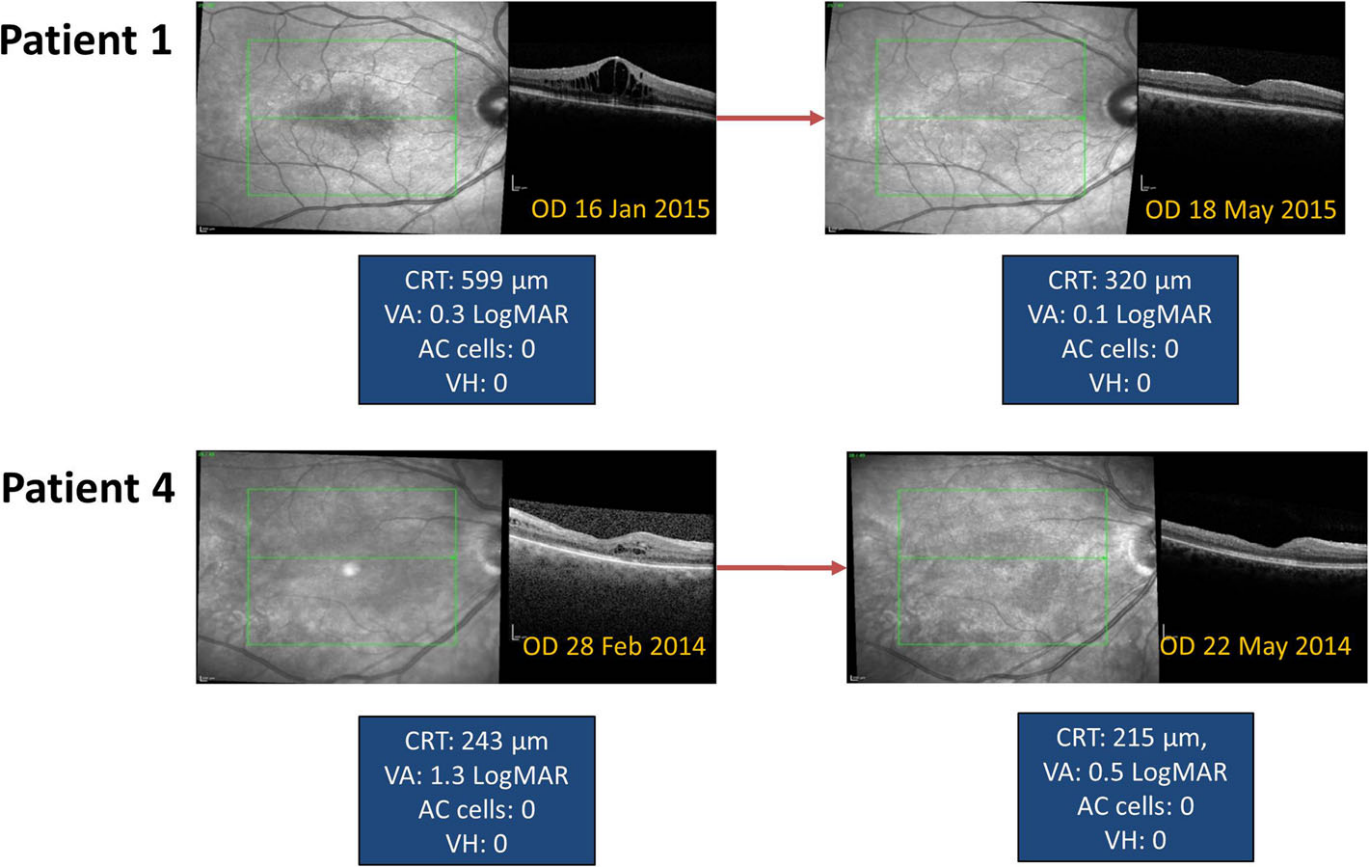
Optical coherence tomography (OCT) images of the macula showing initial improvement in central retinal thickness (CRT) and visual acuity (VA) within the first few months of injecting a single 0.2 μg/day FAc intravitreal implant [9]. **Abbreviations:** OD, right eye; CRT, central retinal thickness; VA, visual acuity; MAR, minimum angle of resolution. Images from Patient 1 are modified from Weber et al. J Ophthalmic Inflamm Infect 2019; 9: 38 and are reproduced under the terms of the Creative Commons Attribution 4.0 International License (http://creativecommons.org/licenses/by/4.0/). Copyright lies with the authors of Weber et al. [10]

**Fig. 5:**
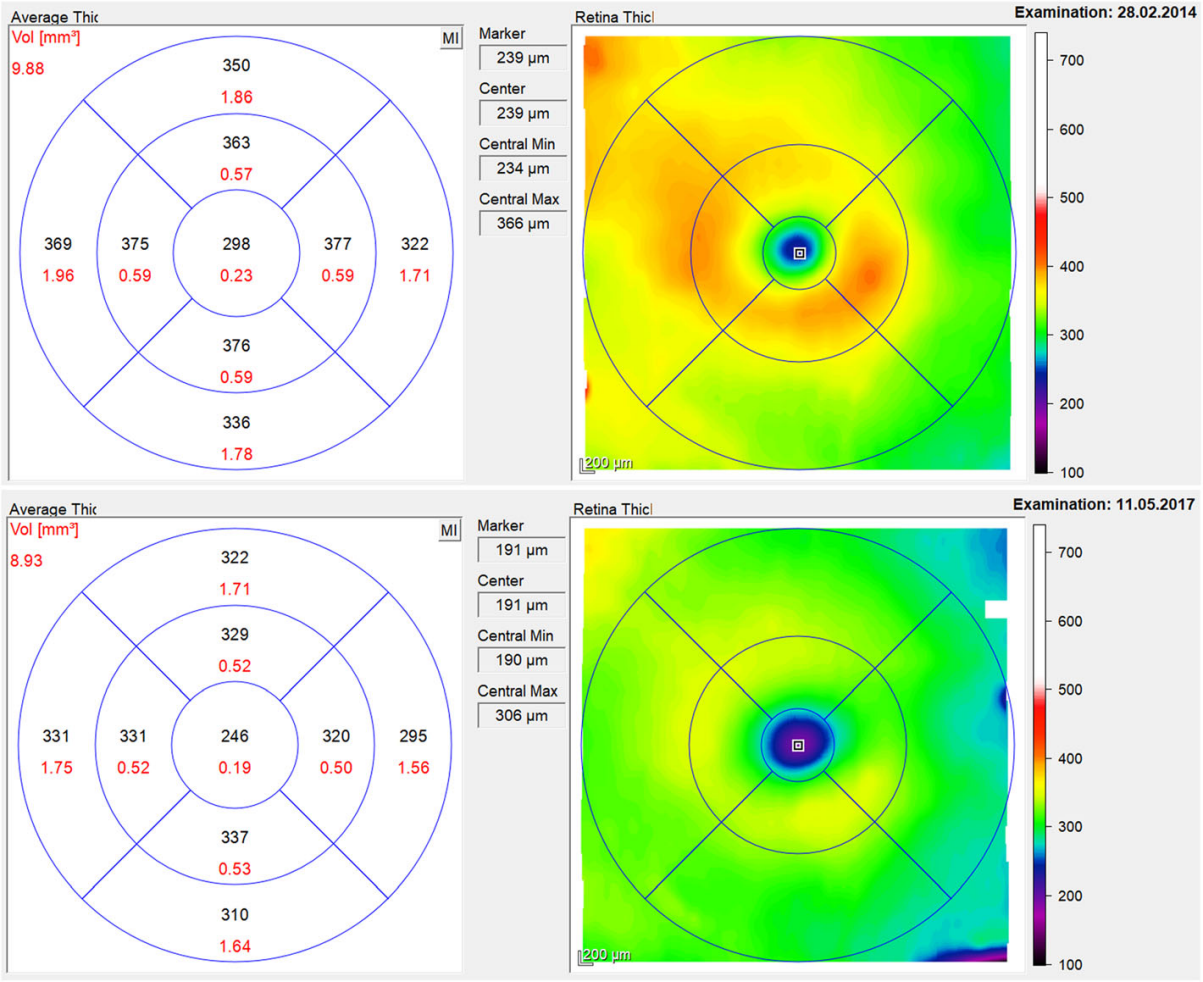
Macular topographic maps (left panels) and colour macular maps (right) to show the sustained reduction in central retinal thickness for more than 3 years after treatment with a single 0.2 μg/day FAc intravitreal implant in a patient with non-infectious intermediate uveitis. Images shown for a single patient eye where the FAc implant led to a sustained thinning of retinal thickness to below 250 μm (i.e. a dry retina) throughout the period between the 28th February 2014 and the 11th May 2017. During this period the eye did not require re-treatment until 42 months after receiving the first FAc implant. The patient had previously received therapy for chronic macular oedema for 4 years including multiple intravitreal triamcinolone or dexamethasone implants and systemic therapies (details of this patient are reported as case 4 in Weber et al. 2019 [10]

The 0.2 μg/day FAc implant was first approved for the treatment of diabetic macular oedema a few years ago [1], so off-label use of the implant in uveitis has been possible for some time. A retrospective evaluation of a case series of 11 eyes (from 8 patients) with non-infectious uveitic macular oedema shows that treatment with the FAc implant led to overall improvements in central retinal thickness (CRT) and visual acuity, and a reduction in uveitic activity (Fig. 4) [10]. All the eyes showed an initial improvement in CRT with a mean (± SD) maximum decrease of 168 ± 202 μm. In addition, a dry macula was achieved in 73% of eyes. A reduction in CRT was associated with an improvement in visual acuity, and 82% of eyes showed at least a 1-line improvement in visual acuity at 6 months post-FAc implantation. Furthermore, 82% of eyes showed reduced or inactive inflammation at the end of the follow-up period (a median of 19 months, range 8–42 months). One of the eyes did not require re-treatment for 42 months. Figure 5 shows macular topographic maps and colour macular maps to show the sustained reduction in central retinal thickness (to below 250 μm) for more than 3 years (i.e. between the 28th February 2014 and the 11th May 2017) after treatment with a single 0.2 μg/day intravitreal implant in a patient with non-infectious intermediate uveitis. It appears that the duration of effect of the implant varies between patients, with some not requiring re-treatment for considerably longer than 3 years while others may require re-treatment earlier than this.

Overall, 9.1% (1/11) of eyes showed an increase in intraocular pressure of more than 10 mmHg and 0% showed an increase of more than 30 mmHg. None of the eyes required additional topical medication or surgery to lower intraocular pressure. Two of the eyes were phakic at baseline and both developed cataracts and required surgery. No major complications were reported (including no endophthalmitis, hypotony, retinal detachment or implant dislocation).

In summary, these real-world data show that the FAc implant is a valuable and long-lasting treatment for non-infectious uveitic macular oedema. The long duration of action of the FAc implant minimises the number of injections needed and so may reduce the burden of treatment compared with other therapeutic approaches — which is valuable for patients wishing to minimise the number of injections they receive, particularly needle-phobic patients. The FAc implant has an acceptable safety profile and can help to minimise the potential for systemic adverse events (because its localised intravitreal delivery of corticosteroid into the posterior segment of the eye can obviate the need for systemic therapy).

#### Key point from presentation

The 0.2 μg/day FAc intravitreal implant offers a systemic therapy-sparing treatment option for non-infectious uveitis affecting the posterior segment. The FAc implant offers efficacy for up to 3 years and so may reduce the burden of treatment by minimising the number of injections needed.

### Case presentations – retinal vasculitis and birdshot retinochoroiditis by Mr. Carlos Pavesio

**Fig. 6:**
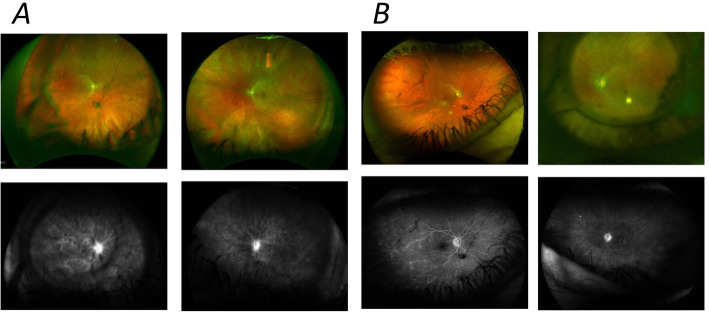
Angiograms from patient with retinal vasculitis before **a** and in excess of 3 years after treatment with the 0.2 μg/day FAc intravitreal implant **b**. Left panels: right eye; right panels, left eye; top panels, wide-field colour fundus photos; bottom panels, fundus fluorescein angiography. Images in Fig. 6 A were taken in July 2015. The patient was treated bilaterally with ILUVIEN in January and February 2016 (right and left eyes, respectively) and the images in Fig. 6, obtained in September 2019, were obtained 3 years after treatment with the 0.2 μg/day FAc implant

The 0.2 μg/day FAc intravitreal implant has been used in a patient with retinal vasculitis. The 58-year-old white female had floaters in both eyes, normal anterior segments and normal intraocular pressure. Angiograms showed marked vascular leakage, vitreous opacities and macular oedema. She did not tolerate oral steroids well and had a poor response to mycophenolate mofetil. She was treated with the 0.2 μg/day FAc implant in both eyes and, 3 years later, she was still continuing to benefit — maintaining both good control of the inflammatory process, as demonstrated by the absence of active leakage in a fluorescein angiography, and good vision (Fig. 6).

The 0.2 μg/day FAc intravitreal implant has also been used in a patient with birdshot retinochoroiditis (HLA-A29+). Patients with this condition tend to strongly dislike the medications they are given and so are generally very receptive to local therapy.

**Fig. 7:**
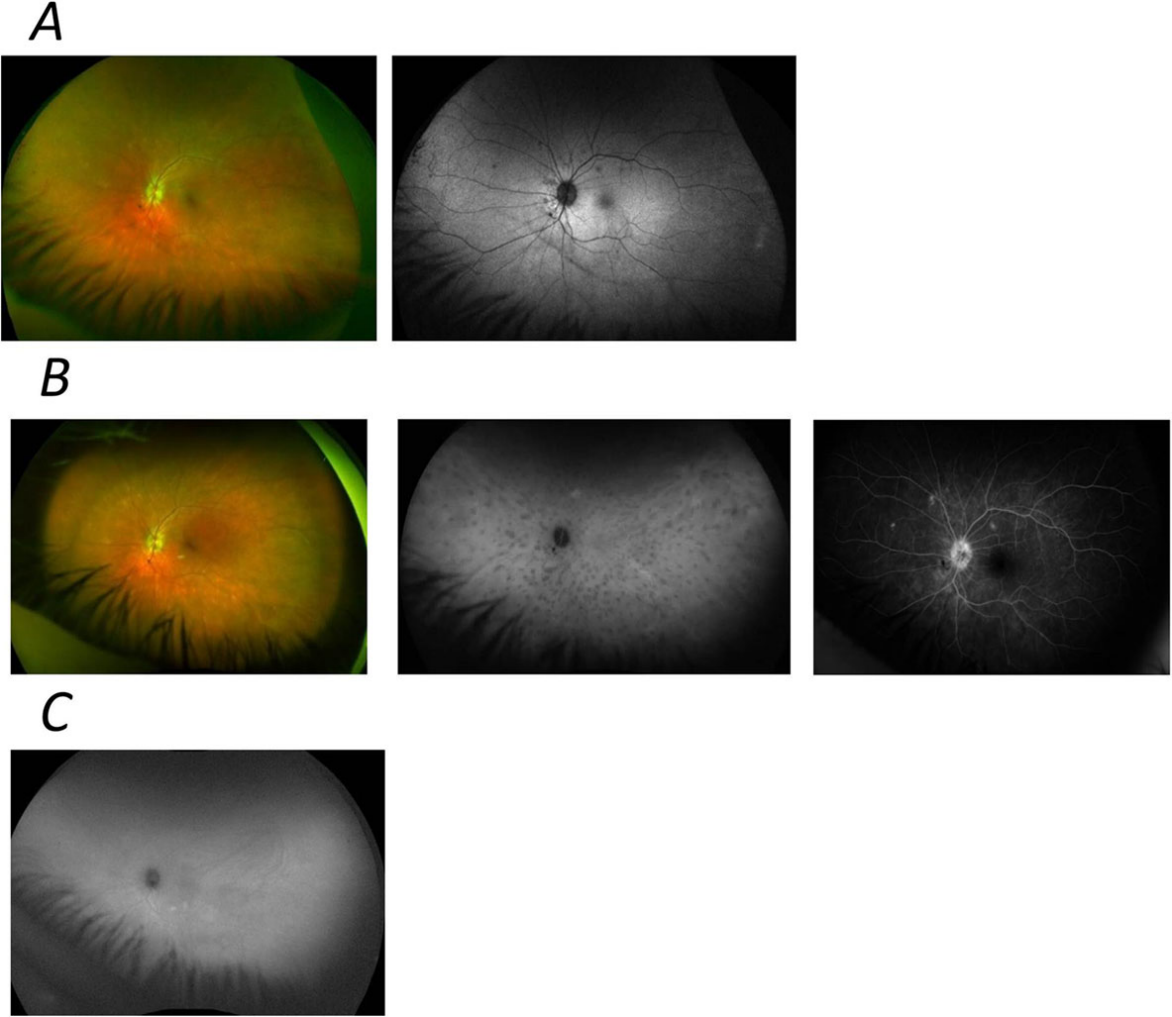
Angiograms from a patient with birdshot retinochoroiditis before **a** and after being treated with the 0.2 μg/day FAc intravitreal implant **b** before then also being treated later with adalimumab **c**. Figure 7 **a** shows images of the left eye in colour fundus (left panel) and fundus fluorescein angiography (right) in March 2016. Figure 7 **b** shows images taken in June 2016 after treatment with the FAc implant and shows no change in the choroidal lesions. Figure 7**c** shows complete resolution of choroidal dark spots in May 2019. At this point adalimumab had also been given (administered in January 2019)

Both eyes were clinically quiet with visual acuity of 6/5. They showed vitreous involvement, with some degree of vitreous opacity and some mild vascular leakage, but no significant macular oedema. The patient was very disturbed by the symptoms related to the vitreous involvement and, without medication, these tended to get worse. As birdshot retinochoroiditis affects the retina generally, and is not localised to the macula, patients can have a reduction in retinal function even when their central vision may be preserved until very late in the disease. The patient did not tolerate oral steroids, tacrolimus or mycophenolate mofetil and so both eyes were treated with the 0.2 μg/day FAc intravitreal implant (Fig. 7).

This resulted in clearing of the vitreous and the patient was very satisfied. However, the choroidal lesions remained unchanged, the choroid was thick and retinal pigment epithelium damage was developing in the peripapillary area. Although the retinal vasculature was controlled by the implant, the choroidal inflammation was beginning to damage the outer retina. To address this, adalimumab therapy was started. This improved angiographic findings and the choroidal dark spots improved progressively until they were completely resolved (Fig. 7). After 7 weeks of adalimumab therapy there was also a significant reduction in choroidal thickness.

This unpublished case illustrates the clinical benefit of the FAc implant in retinal vascular disease but also shows that it is not effective for choroidal pathology. Nevertheless, it demonstrates that combination therapy can be a highly successful approach to treating birdshot retinochoroiditis — with the FAc implant used to control the vascular retinal component of the disease and systemic therapy used to control the choroidal pathology.

#### Key point from presentation

In case histories, the 0.2 μg/day FAc intravitreal implant has demonstrated efficacy in the treatment of retinal vasculitis and birdshot retinochoroiditis.

## Conclusions

Chronic or recurrent uveitis needs a long-acting treatment to minimise the potential for repeated cycles of inflammation that cause progressive retinal damage. The 0.2 μg/day FAc intravitreal implant offers a systemic therapy-sparing treatment option to prevent recurrence of NIU-PS, and is designed to release a low daily dose of corticosteroid into the vitreous for up to 3 years.

Results from an early investigational new drug study performed through the US FDA complement those from subsequent phase 3 data and show that the FAc implant has a long-lasting effect — among 12 patients with chronic recurrent non-infectious uveitis, 42% had no recurrence of uveitis or cystoid macular oedema during the follow-up period of between 36 and 80 months. In the remaining patients, recurrence of cystoid macular oedema occurred after a mean of 36.9 months and recurrence of uveitis occurred after a mean of at least 29.8 months.

Phase 3 data show that, in the 36 months post-treatment and compared with a sham injection, treatment with the FAc implant is associated with significantly fewer episodes of uveitic recurrence, a significantly longer time to uveitic recurrence, greater improvement in visual acuity, a lower need for adjunctive therapy, and an acceptable safety profile.

In addition to these impressive results in non-infectious uveitis that are unmatched by other currently available treatments, initial reports from case histories also suggest the 0.2 μg/day FAc intravitreal implant may be effective in the treatment of retinal vasculitis and birdshot retinochoroiditis.

## Data Availability

Please contact authors for data requests.

## Abbreviations

CRT: Central retinal thickness
FAc: Fluocinolone acetonide
FDA: Food and Drug Administration
HLA-A29: Birdshot retinochoroiditis
IOP: Intra-ocular pressure
MAR: Minimum angle of resolution
NIU-PS: Non-infectious uveitis affecting the posterior segment of the eye
OD: Right eye
SD: Standard deviation
VA: Visual acuity
